# Effectiveness of historical smallpox vaccination against mpox clade II in men in Denmark, France, the Netherlands and Spain, 2022

**DOI:** 10.2807/1560-7917.ES.2024.29.34.2400139

**Published:** 2024-08-22

**Authors:** Soledad Colombe, Silvia Funke, Anders Koch, Manon Haverkate, Susana Monge, Anne-Sophie Barret, Aisling Vaughan, Susan Hahné, Catharina van Ewijk, Hanne-Dorthe Emborg, Sebastian von Schreeb, Asunción Díaz, Carmen Olmedo, Laura Zanetti, Daniel Levy-Bruhl, Luis Alves de Sousa, José Hagan, Nathalie Nicolay, Richard Pebody

**Affiliations:** 1 World Health Organization Regional Office for Europe, Copenhagen, Denmark; 2 Institute of Tropical Medicine, Antwerp, Belgium; 3 European Centre for Disease Prevention and Control (ECDC), Stockholm, Sweden; 4 Infectious Disease Epidemiology and Prevention, Statens Serum Institut, Copenhagen, Denmark; 5 Department of Infectious Diseases, Rigshospitalet University Hospital, Copenhagen, Denmark; 6 Centre for Infectious Disease Control, National Institute for Public Health and the Environment (RIVM), Bilthoven, the Netherlands; 7 National Centre of Epidemiology, Carlos III Institute of Health, CIBERINFEC, Madrid, Spain; 8 Santé Publique France, St-Maurice, France; 9 European Programme for Intervention Epidemiology Training (EPIET), European Centre for Disease Prevention and Control (ECDC), Stockholm, Sweden; 10 Department of Infectious Diseases, Copenhagen University Hospital - Amager and Hvidovre Hospital, Copenhagen, Denmark; 11 Vaccination Programme, Ministry of Health, Madrid, Spain

**Keywords:** Europe, mpox, vaccine effectiveness, monkeypox, childhood smallpox vaccination, screening method

## Abstract

**Background:**

In 2022, a global monkeypox virus (MPXV) clade II epidemic occurred mainly among men who have sex with men. Until early 1980s, European smallpox vaccination programmes were part of worldwide smallpox eradication efforts. Having received smallpox vaccine > 20 years ago may provide some cross-protection against MPXV.

**Aim:**

To assess the effectiveness of historical smallpox vaccination against laboratory-confirmed mpox in 2022 in Europe.

**Methods:**

European countries with sufficient data on case vaccination status and historical smallpox vaccination coverage were included. We selected mpox cases born in these countries during the height of the national smallpox vaccination campaigns (latest 1971), male, with date of onset before 1 August 2022. We estimated vaccine effectiveness (VE) and corresponding 95% CI for each country using logistic regression as per the Farrington screening method. We calculated a pooled estimate using a random effects model.

**Results:**

In Denmark, France, the Netherlands and Spain, historical smallpox vaccination coverage was high (80–90%) until the end of the 1960s. VE estimates varied widely (40–80%, I^2^ = 82%), possibly reflecting different booster strategies. The pooled VE estimate was 70% (95% CI: 23–89%).

**Conclusion:**

Our findings suggest residual cross-protection by historical smallpox vaccination against mpox caused by MPXV clade II in men with high uncertainty and heterogeneity. Individuals at high-risk of exposure should be offered mpox vaccination, following national recommendations, regardless of prior smallpox vaccine history, until further evidence becomes available. There is an urgent need to conduct similar studies in sub-Saharan countries currently affected by the MPXV clade I outbreak.

**Key public health message**

**What did you want to address in this study and why?**

Following the eradication of smallpox 40 years ago, routine smallpox vaccination ended, leading to a growing proportion of the population in Europe susceptible to monkeypox virus (MPXV) and other Orthopoxviruses. With the recent surge in mpox cases globally, we sought to determine the effectiveness of historical smallpox vaccination against mpox caused by MPXV clade II with the aim to inform ongoing mpox vaccination policies.

**What have we learnt from this study?**

Our analyses revealed that in a European setting, more than two-thirds of men who were vaccinated against smallpox during childhood, are likely to retain some protection against mpox caused by MPXV clade II. However, the degree of protection varied widely among the four countries we investigated, likely due to the differences in smallpox vaccination schedules and further research is required to validate these findings more conclusively.

**What are the implications of your findings for public health?**

Our findings suggest that individuals at higher risk of MPXV clade II infection should be offered mpox vaccination, in line with national recommendations, regardless of prior smallpox vaccine history, until further evidence becomes available to inform future mpox vaccination strategies.

## Introduction

In 2022, a global outbreak of mpox caused by monkeypox virus (MPXV) clade II emerged, mostly affecting young adult men reporting having sex with men [1]. Monkeypox virus, the virus that causes mpox disease, belongs to the same genus as the smallpox virus i.e. the Orthopoxvirus genus in the Poxviridae family. Unlike smallpox, the geographical distribution of mpox was historically limited to Central and West Africa, where small outbreaks of zoonotic origin have been reported [2].

In 2003, the first outbreak outside Central and West Africa was detected in the United States (US), and between 2003 and 2022, several small clusters and outbreaks were reported outside endemic areas [2]. The 2022 worldwide outbreak raised many questions about possible residual cross-protection of smallpox vaccination for individuals vaccinated during smallpox eradication programmes several decades ago [1,3].

Most smallpox vaccination programmes in Europe started in the early 20th century with vaccinia-based vaccines [4] and mainly targeted children less than 3 years of age for primary vaccination. In some countries this was complemented with booster vaccination strategies in adolescents and/or young adults. This, together with intensified case finding and contact tracing, led to smallpox elimination in the World Health Organization (WHO) European Region in 1953 [4]. However, as part of global efforts to eradicate the disease and the WHO Intensified Eradication plan (1967), high vaccination rates were still observed in the WHO European Region until the mid-1960s, after which uptake decreased rapidly until the early 1980s when smallpox was officially declared eradicated worldwide [4]. In Denmark, France, the Netherlands and Spain, countries of focus in this study, smallpox vaccination campaigns stopped in 1977, 1979, 1976 and 1979, respectively [5-8].

The increase in frequency, size and geographical spread of mpox outbreaks since routine smallpox vaccination ended in 1980 support the hypothesis that childhood smallpox vaccination programmes may provide some protection against MPXV infection and mpox [4,9-12]. The that lack of vaccination since the 1980s might then result in an increasing proportion of the population being susceptible to MPXV and other Orthopoxviruses in Africa and elsewhere [2,9].

Studies in the Democratic Republic of the Congo conducted more than 30 years ago estimated that the vaccinia-based smallpox vaccine was more than 80% effective in preventing mpox and reduced disease severity for MPXV clade I [9,10,13,14]. Two immunogenicity studies conducted during the US outbreak of MPXV clade II in 2003 also suggested possible residual protection, although incomplete, from childhood smallpox vaccination [15,16]. In addition to limited vaccine supply, these results were used as a rationale to prioritise the distribution of vaccine doses during the 2022–2023 mpox outbreak based on prior vaccination status of the target persons and to only provide one dose to those previously vaccinated in many European countries [17-21].

In June 2022, the European Medicine Agency (EMA) authorised the use of Imvanex (Modified vaccinia Ankara – Bavarian Nordic or MVA-BN) under exceptional circumstances for the prevention of mpox. The MVA-BN vaccines are currently authorised for use against infection and disease caused by both smallpox and MPXV in the US (JynneosTM) and Canada (ImvamuneTM) as well as other related Orthopoxviruses (Canada only). These vaccines are third-generation replication-deficient smallpox vaccines [22]. Investigation of the protection offered by recent pre-exposure vaccination with MVA-BN vaccines against MPXV clade II infection during the 2022–2023 epidemic in Europe and the US showed encouraging results. Vaccine effectiveness (VE) of two pre-exposure vaccine (PPV) doses was estimated at between 66% and 89%, while even one PPV dose provided effectiveness between 36% and 86% [23-28].

Residual effectiveness of vaccination with first- and second-generation smallpox vaccines administered several decades ago against infection and severe disease during the 2022­–2023 mpox outbreak is, however, unclear, especially in Europe. Analyses conducted among mpox cases from May 2022 up to August 2022 in the Netherlands suggested that smallpox vaccination during childhood provides a VE against moderate/severe mpox of 58% (95% confidence interval (CI): 15–80%) after adjusting for age [29]. Other studies conducted in Spain, France and within the WHO European Region failed to show a significant association between prior smallpox vaccination and development of complications or hospitalisation due to mpox [3,30,31]. To our knowledge, no study on VE of historical smallpox vaccination against mpox, regardless of severity, has been undertaken to date in Europe.

As at 7 December 2023, a total of 26,112 laboratory-confirmed mpox cases caused by MPXV clade II had been reported in The European Surveillance System (TESSy) by 45 countries and areas in the WHO European Region. Although the peak of the epidemic occurred during the summer of 2022, cases continued to be reported, with 285 cases from 18 countries reported in TESSy between 1 September 2023 and 7 December 2023. Overall, 15% (1,024/6,825) of reported cases with information on prior smallpox vaccination were recorded as having been vaccinated before 2022, with a median age of 49 years (interquartile range (IQR): 39–56) among those previously vaccinated compared with 35 years (IQR: 30–41) among those not previously vaccinated.

In this study, we aimed to estimate the VE of historical smallpox vaccination against laboratory-confirmed mpox caused by MPXV clade II during the 2022–2023 outbreak in Europe to guide future mpox vaccination policies and campaign rollout. Results from this study could help to further inform vaccine recommendations for future emerging outbreaks of Orthopoxviruses.

## Methods

### Context description and case selection

Since March 2022, data on mpox cases were submitted via a case report form by all the countries and areas of the WHO European Region to the European Centre for Disease Prevention and Control (ECDC) and WHO Regional Office for Europe through The European Surveillance System (TESSy) database hosted at ECDC.

We selected countries known to have available information on prior smallpox vaccination status of cases and national historical smallpox vaccination coverage, namely Denmark, France (mainland only), the Kingdom of the Netherlands excluding Dutch Caribbean (called the Netherlands throughout the rest of this manuscript), and Spain. We extracted case-based mpox data submitted to TESSy from these four countries, which was further complemented with supplementary information by countries.

We restricted the analysis to cases recorded as male in TESSy, as too few female cases (less than 2.3%) had been reported to be able to adjust for sex in the analysis. We excluded cases born abroad or born after the peak of the national smallpox vaccination programme in each country in order to include cases that were most likely eligible to be part of the smallpox vaccination programmes in their country. Additionally, we only included cases with date of onset (i.e. symptom onset or, if asymptomatic or missing date, earliest date of either reporting or laboratory sampling) before 1 August 2022, by when most countries had initiated fully running pre-exposure and post-exposure vaccination programmes. This was done to avoid vaccination during the mpox outbreak as a confounding factor. We further excluded any case that had received a dose of MVA-BN vaccine more than a week before date of onset.

### Study design

The screening method, as proposed by Farrington [32], estimates VE in a simple and rapid way using two sources of data: (i) historical vaccination coverage data in the reference population, and (ii) information on case vaccination status using information collected for disease surveillance. This approximates the VE as 1 – OR (odds ratio) of vaccination in cases to vaccination coverage in the reference population [32].

#### Ascertainment prior to smallpox vaccination status of cases

In Denmark, case vaccination status was self-reported to the diagnosing clinician, and cases were contacted in person by telephone by the study group to corroborate the records. In France, vaccination status was self-reported by cases to the epidemiological investigation team through phone interviews. In the Netherlands and Spain, vaccination status was self-reported to the diagnosing clinician, and in some cases the presence of a scar was checked.

#### Vaccine coverage in the reference population

The specificities of the smallpox vaccine programmes in the four countries are described in Table 1.

**Table 1:** Summary of smallpox vaccination programmes in the reference populations, Denmark, France, the Netherlands, Spain, 1900–1984

	Denmark [5,46]	France [6]	The Netherlands [7,18]	Spain [8]
Beginning of smallpox vaccination	1931	1901	1900	1900
End of smallpox vaccination	1977	1979 for primary vaccination 1984 for booster doses	1976	1979
Target population of primary vaccination	Children before school admission	Children during the first year of life	Children under 1 year of age (but regularly given up to 2 years of age)	Children at 20 months of age, with catch-up doses administered at school age or later (e.g. military service)
Booster doses	No	Yes, boosters recommended at ages 11 and 21 years (incl. during mandatory military service)	No (rarely in the military)	Yes, for men in the military service
Mandatory	Yes, before school admission, except when immunocompromised	Yes, for primary vaccination and military service	Yes, for school children until 1928	No

Vaccine coverage in the Danish reference population was obtained from a study by Sørup et al. [33]. In this study, vaccination data were obtained from school registers between 1965 and 1977 in Copenhagen, Denmark, during which period smallpox vaccination in Denmark was being phased out. It was assumed that vaccination coverage before 1965 was stable and equal to vaccination coverage in 1965.

Vaccine coverage in France was calculated using archived population data from a national census (National Institute of Statistics and Economic Studies), and annual reports on number of children vaccinated per year from the National Institute of Health and Medical Research, which did not include booster doses [6]. However, for the vast majority of men born up until and including 1962, a booster was likely administered during military service.

Vaccine coverage in the Netherlands was assessed through number of doses administered in the country. Number of doses administered per year were only available from 1931 onwards and we assumed coverage before that was the same as in 1931.

Vaccine coverage in Spain was calculated using number of doses administered per year [8] divided by the number of children in target age groups living in Spain at the time (Statistics National Institute), and assuming some of the doses were administered at school age or later (between 2% and 10% depending on the year). Estimation of vaccine coverage did not include booster doses.

We assumed there was no difference in population coverage between boys and girls for vaccination campaigns targeting children.

### Statistical analysis

Using Farrington’s screening method [32], we calculated VE estimates and their 95% CIs for each of the four countries using logistic regression, with the case vaccination status as dependent variable and the logit of the vaccination coverage in the reference population as an offset. In addition, we calculated a pooled estimate with a random effects approach using the Paule-Mandel method [34]. A random effects approach was used to account for large differences in vaccine programme implementation across all four countries. We restricted all analyses to birth cohorts born during the height of the national smallpox vaccination campaigns (before their phasing out), namely before 1967 (Denmark), 1966 (France), 1971 (the Netherlands) and 1970 (Spain) (see Results section). We conducted two sensitivity analyses where we considered all cases with missing information on prior smallpox vaccination as having been vaccinated or as not having been vaccinated, respectively. All statistical analyses were conducted in R version 4.3.0 [35]. The random effects model was built using the R package meta version 6.2–1.

## Results

**Figure 1:**
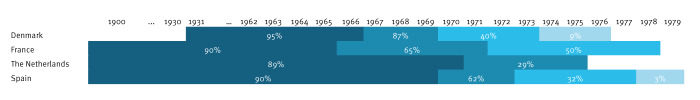
Estimates of smallpox vaccination coverage^a^ by birth cohort, Denmark, France, the Netherlands, and Spain, 1900–1979

^a^ Coverage estimates are distinguished by the colour intensity. Lighter shades of blue represent lower coverage.

In all four countries, estimates of historical smallpox vaccination coverage were high (80–90%) and stable until the end of the 1960s, but then reduced considerably during the past 10 years of the vaccination programmes (Figure 1). The period of the vaccination programmes used for vaccination coverage estimates is indicated in dark blue on Figure 1. It corresponds in each country to the period of high vaccine coverage (called height of the vaccination campaign in this study).

**Figure 2:**
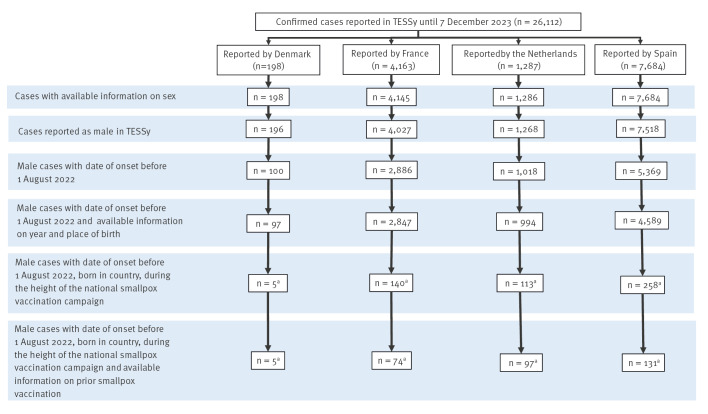
Flow of case selection for inclusion in the analysis, Denmark, France, the Netherlands and Spain

As at 7 December 2023, a total of 198 laboratory-confirmed mpox cases had been reported in Denmark, 4,163 in France, 1,287 in the Netherlands and 7,684 in Spain of any age and sex. Most of them (98%) were male (196/198 in Denmark, 4,027/4,145 in France, 1,268/1,286 in the Netherlands and 7,518/7,684 in Spain). Case selection is detailed in Figure 2.

TESSy: The European Surveillance System.

^a^ Denmark before 1967, France before 1966, the Netherlands before 1971, Spain before 1970.

Cases included in the analysis had a median age of 60 (IQR: 58–60), 60 (IQR: 58–63), 57 (IQR: 53–62) and 52 (IQR: 46–48) years in Denmark, France, the Netherlands and Spain, respectively.

Estimates of the VE of prior smallpox vaccination against mpox for men born during the height of the national smallpox vaccination campaigns varied widely between countries, ranging from 42% to 84% (Table 2).

**Table 2:** Country-specific estimates of historical smallpox vaccine effectiveness (VE) against mpox, Denmark, the Netherlands, France and Spain, March–July 2022

Country	Birth cohort^a^	Number of cases with vaccination information	Proportion of the population vaccinated	Number of cases vaccinated	Proportion of vaccinated cases (%)	VE (95% CI)
Denmark	Born up to and including 1966	5	95%	5	100	NA (NA–100)
France	Born up to and including 1965	74	90%	54	73	70% (50–82)
The Netherlands	Born up to and including 1970	97	89%	80	82	42% (2–66)
Spain	Born up to and including 1969	131	90%	78	60	84% (77–88)

CI: confidence interval; NA: not ascertainable; VE: vaccine effectiveness.

^a^ Birth cohorts from before the smallpox vaccination programme was phased out in each country.

**Figure 3:**
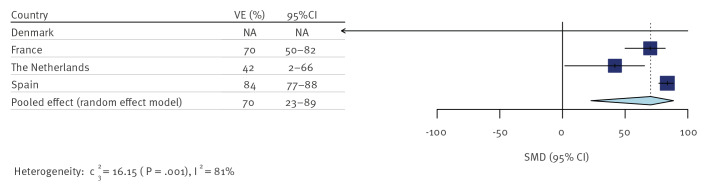
Historical smallpox vaccine effectiveness against mpox pooled over all countries, Denmark^a^, France, the Netherlands and Spain, March–July 2022

CI: confidence interval; NA: not applicable; SMD: standardised mean difference; VE: vaccine effectiveness.

^a^ The weight of Denmark’s estimate was 0% due to high uncertainty in the true within-country variance.

The pooled VE estimate restricted to birth cohorts from the height of the vaccination phase was 70% (95% CI: 23–89%) and showed high heterogeneity between single estimates (I^2^ = 82%) (Figure 3). The weight of estimates from France (32.4%), the Netherlands (32.2%) and Spain (35.4%) in the pooled analysis was similar, while the weight of Denmark’s estimate was 0% due to a very small number of cases and high uncertainty in the true within-country variance.

The sensitivity analyses (Table 3) showed that the estimate of VE varies substantially with changes in the proportion of cases vaccinated, especially when the sample size is small. The point pooled VE estimates varied between 46% and 90%.

**Table 3:** Sensitivity analyses of country-specific estimates and pooled estimates of historical smallpox vaccine effectiveness against mpox in Denmark, the Netherlands, France and Spain, March–July 2022

Country	Denmark	France	The Netherlands	Spain
Total number of cases	5	140	113	258
Cases with known vaccination status	5	74	97	131
Proportion of the population vaccinated	95%	90%	89%	90%
Missing values as non-vaccinated				
Proportion of cases vaccinated	100%	39%	71%	30%
VE (95% CI)	NA (NA–100)	93% (90–95)	70% (54–80)	95% (94–96)
Pooled VE	90% (58–98)			
Missing values as vaccinated				
Proportion of cases vaccinated	100%	86%	85%	79%
VE (95% CI)	NA (NA–100)	33% (-10 to 57)	30% (-21 to 57)	57% (42–68)
Pooled VE	46% (18–65)			

CI: confidence interval; NA: not ascertainable; VE: vaccine effectiveness.

## Discussion

With the drop in smallpox vaccine uptake 50 years ago and the full cessation of vaccination 10 years later, an increasing proportion of the population in Europe is unvaccinated, and therefore is susceptible to mpox and other Orthopoxviruses [11,12]. Our study suggests that an estimated 70% of men vaccinated during the height of the historical smallpox vaccination programmes were likely to remain protected against mpox caused by MPXV clade II, indicating some residual cross-protection, although there is significant heterogeneity in the level of VE between the countries studied. To our knowledge, this study is the first to date to assess VE of smallpox vaccination against mpox regardless of severity in Europe [29,36]. By including cases who had mostly been vaccinated with the smallpox vaccine more than 50 years ago, we could assess the long-term residual effects of prior smallpox vaccination. Another study, conducted in the US in military personnel vaccinated on average 13 years ago showed a similar VE (72–75%) of prior vaccination with first and second-generation smallpox vaccines against recent mpox infection [37]. This supports the hypothesis that in people with prior smallpox vaccination, residual cross-protection remains for a prolonged period, mainly as a result of vaccine-induced cellular immunity [38-42], although there is large uncertainty about the level of protection with large variation in the country-specific estimates.

Military service was compulsory for all men in France and Spain until 1996 and 2001, respectively, and smallpox vaccine coverage during military service was likely extremely high until 1963 in France and 1969 in Spain. In comparison, the vaccination coverage for smallpox vaccination was also high in the military in the Netherlands until 1978, but only a part of all men had to do their service. In Denmark, smallpox vaccination was never offered during military service. It is likely that the higher VE estimates in men in France and Spain are the consequence of boosters given during compulsory military service not only improving coverage in the male population, but also strengthening immunity following a second dose and reducing the time interval between the receipt of the last dose and the current mpox outbreak [38,40,43-45] compared with Denmark and the Netherlands.

In our study, we were not able to distinguish between cases vaccinated as a child, cases vaccinated during military service or cases vaccinated for other reasons such as healthcare or laboratory occupation. We were also not able to retrieve data on number of doses previously received. However, from information on the organisation of the vaccination campaigns in the respective countries, it is highly likely that most French men born up to 1963 and Spanish men born up to 1969 had received at least two doses, while Dutch and Danish cases had received only one dose. These are likely to be important factors in explaining the country differences.

As the majority of reported mpox cases in Europe in 2022 and 2023 were young adult men reporting having sex with men [3], the main risk factor for infection has so far been attributed to high-risk sexual behaviours often attributed to younger age groups [1,3]. However, it is possible that a small part of this observed phenomenon, with lower incidence in older age groups, can be attributed to older men also having been previously vaccinated (unlike the younger men under 42 years of age), and thus being protected against mpox.

Our results need to be interpreted in light of some limitations. There was high heterogeneity in VE estimates between the four countries due to differences in vaccination coverage, including implementation of historical vaccination campaigns, and uncertainty around achieved coverage in specific birth cohorts, which needed to be derived from aggregated numbers of administered doses for three of the four countries. Smallpox vaccination status based on self-reporting or scar check is potentially unreliable [4] and there were a high number of cases with missing information on vaccination status, leading to possible bias in the measurement of the proportion of vaccinated cases, since those vaccinated may be more likely to remember their status than those who are not. Furthermore, sensitivity analyses showed that the VE is very sensitive to assumptions regarding cases with unknown vaccination status. A low number of cases matching the selection criteria in all four countries also led to limited precision around the estimates. Included cases were detected through routine surveillance, possibly implying a bias in case inclusion towards more health-conscious or sicker cases. This study focused on the VE of smallpox vaccination against laboratory-confirmed mpox. If we assume that prior exposure to smallpox vaccination is associated with milder symptoms, then the VE against all MPXV infections (including pauci-symptomatic and asymptomatic cases who would not reach care) would likely be lower. However, within the study population of those reaching care, we expect the VE to be unbiased with respect to clinical severity. Further studies should consider immunological testing of cases across a spectrum of clinical presentations in order to determine possible correlation between prior smallpox vaccination and mpox clinical presentation. In addition, we could not fully ascertain that the cases selected came from the same population that was historically vaccinated. However, it seems unlikely that the vaccine coverage of mpox cases differs from the vaccine coverage of their respective birth cohorts, especially since smallpox vaccination was administered during the first years of life. Furthermore, we used separate population coverage estimates for each country and excluded cases born abroad, which could otherwise have biased the VE estimates.

## Conclusion

Overall, our findings suggest that there is residual cross-protection by historical smallpox vaccination against mpox caused by MPXV clade II in men. However, there is high uncertainty and heterogeneity in the country-specific estimates, making this evidence insufficient to support differential smallpox vaccination to protect against mpox depending on prior childhood vaccination or age. Individuals at high-risk of exposure should be offered mpox vaccination, in line with national recommendations, regardless of prior smallpox vaccine history, until further evidence becomes available. There is an urgent need to conduct similar studies on smallpox vaccine effectiveness against MPXV clade I in Sub-Saharan countries currently affected by the MPXV clade I outbreak, including female if sample size allows.
