# Author’s correction for Euro Surveill. 2024;29(34)

**DOI:** 10.2807/1560-7917.ES.2024.29.39.2409269

**Published:** 2024-09-26

**Authors:** Eurosurveillance editorial team

**Affiliations:** 1 European Centre for Disease Prevention and Control (ECDC), Stockholm, Sweden

In the article entitled ‘Effectiveness of historical smallpox vaccination against mpox clade II in men in Denmark, France, the Netherlands and Spain, 2022’ by Colombe et al., published on 22 August 2024, affiliation ‘9. European Programme for Intervention Epidemiology Training (EPIET), European Centre for Disease Prevention and Control (ECDC), Stockholm, Sweden’ was removed for Sebastian von Schreeb on 26 Sep 2024 at the request of the authors.

